# Peptidylarginine Deiminase 2 in Host Immunity: Current Insights and Perspectives

**DOI:** 10.3389/fimmu.2021.761946

**Published:** 2021-11-04

**Authors:** Zhenyu Wu, Patrick Li, Yuzi Tian, Wenlu Ouyang, Jessie Wai-Yan Ho, Hasan B. Alam, Yongqing Li

**Affiliations:** ^1^Department of Surgery, University of Michigan Hospital, Ann Arbor, MI, United States; ^2^Department of Infectious Diseases, Xiangya 2^nd^ Hospital, Central South University, Changsha, China; ^3^Department of Internal Medicine, New York University (NYU) Langone Health, New York, NY, United States; ^4^Department of Rheumatology, Xiangya Hospital, Central South University, Changsha, China; ^5^Department of Surgery, Feinberg School of Medicine, Northwestern University, Chicago, IL, United States

**Keywords:** PAD2, autoimmune diseases, sepsis, NETosis, pyroptosis

## Abstract

Peptidylarginine deiminases (PADs) are a group of enzymes that catalyze post-translational modifications of proteins by converting arginine residues into citrullines. Among the five members of the PAD family, PAD2 and PAD4 are the most frequently studied because of their abundant expression in immune cells. An increasing number of studies have identified PAD2 as an essential factor in the pathogenesis of many diseases. The successes of preclinical research targeting PAD2 highlights the therapeutic potential of PAD2 inhibition, particularly in sepsis and autoimmune diseases. However, the underlying mechanisms by which PAD2 mediates host immunity remain largely unknown. In this review, we will discuss the role of PAD2 in different types of cell death signaling pathways and the related immune disorders contrasted with functions of PAD4, providing novel therapeutic strategies for PAD2-associated pathology.

## Highlights

- Peptidylarginine deiminase (PAD) enzymes catalyze the conversion of arginine residues to citrulline, regulating activity of host immunity.
- PAD2 plays an important yet different role in immune cells than its isozyme PAD4. Although PAD4 is previously identified to be the key regulator in the formation of neutrophil extracellular traps (NETosis), PAD2 also takes part in NETosis in the absence of PAD4.
- *Pad2* deficiency decreases macrophage pyroptosis while *Pad4* deficiency increases pyroptosis.
- PAD2, differing from the other PAD family members, citrullinates arginine 1810 (Cit1810) in repeat 31 of the carboxyl-terminal domain of the largest subunit of RNA polymerase II, which enables the efficient transcription of highly expressed genes needed for cell cycle progression, metabolism, and cell proliferation.

## Introduction

**Figure 1:**
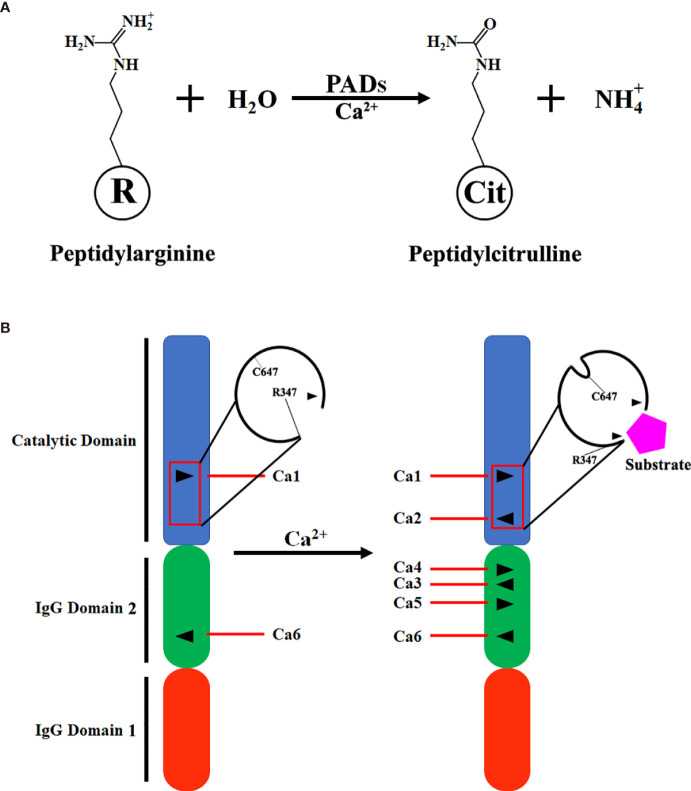
Scheme of citrullination and PAD2 structure. **(A)** A simplified equation describing that PAD2 catalyzes the citrullination of a peptidylarginine residue in the presence of calcium ions (Ca^2+^). **(B)** Transition of inactivated PAD2 to activated PAD2. Ca1 and Ca6 in PAD2 protein are permanently occupied by Ca^2+^; when the levels of Ca^2+^ in cytoplasm are elevated, Ca2, Ca3, Ca4 and Ca5 are filled with Ca^2+^ for PAD2 activation. Ca1, Ca2, Ca3, Ca4, Ca5, and Ca6, Calcium-binding sites 1, 2, 3, 4, 5, and 6; IgG, immunoglobin; PAD, peptidylarginine deiminase.

Peptidylarginine deiminases (PADs) are a group of enzymes that catalyze post-translational modification of proteins by converting arginine residues into citrullines (**Figure 1A**) (1, 2). The PAD family consists of five members: PAD1, PAD2, PAD3, PAD4, and PAD6 (3). As the most widely expressed member, PAD2 can be found in many tissues and organs, including brain (4), spinal cord (4), spleen (5), pancreas (6), skeletal muscles (7), secretory glands, and immune cells (8, 9). By citrullinating proteins, PAD2 regulates a number of cellular processes such as gene transcription (10, 11), antigen generation (12), extracellular trap formation (also termed ETosis) (13, 14), and pyroptosis (15).

ETosis is a described cell death that results in the release of a complex lattice of chromatin containing DNA, histones, and other associated proteins (16–18). These extracellular chromatin webs can entrap and kill microbial organisms. Originally, this phenomenon was described in neutrophils, termed NETosis (Neutrophil Extracellular Traps). However, researchers later found that this mechanism also exists in other cell types such as macrophages, eosinophils, and mast cells (19). Thus, some researchers recommend that the mechanism of this cell death be generalized as “ETosis” (20–22), while others prefer using NETosis or macrophage ETosis (METosis) for the death of specific cell sources.

Similar to the structure of PAD4 (23), the N-terminal of PAD2 consists of two immunoglobulin-like domains, IgG domain 1 (residues 1-115) and IgG domain 2 (residues 116-295), and a catalytic domain, the C-terminal (residues 296-665) (24). There are six calcium-binding sites in PAD2 (Ca1-6). Ca1 and Ca6 are occupied by calcium in both inactivated and activated PAD2. During the activation of PAD2, calcium ions bind to sites Ca3-5. Afterwards, calcium binds to Ca2, which causes conformational changes at the active site. R347 moves out of the active site, and C647 moves in. As such, a pocket-like structure is generated for substrate binding (**Figure 1B**). Then, where does the calcium come from for PAD2 activation? A previous study revealed that adenosine triphosphate (ATP)-induced PAD2 activation can be dramatically diminished in mast cells cultured in calcium-free media, suggesting that calcium needed for PAD2 activation mainly comes from the extracellular space (25). Zheng et al. also demonstrated that Annexin A5 (ANXA5) can bind to the plasma membrane to facilitate calcium influx and further contribute to PAD2 activation (26). Thus, sufficient extracellular calcium is required for the activation of PAD2.

The substrates of PAD2 are quite diverse *in vivo*, including cell structural proteins (27, 28), immunomodulating molecules (29, 30), and histones (31). For instance, vimentin, which is an important part of the cytoskeleton in skeletal muscles and macrophages, is a PAD2 substrate (28). Another crucial protein for cell structure, actin, can also be citrullinated by PAD2 (27). PAD2 can mediate thrombotic activities *via* citrullinating antithrombin (32) and fibrinogen (33). PAD2-catalyzed citrullination of certain immunomodulating cytokines, such as the chemokine (C-X-C motif) ligand (CXCL) 10 (34), interleukin (IL)-8 (29), and CXCL12 (30) is associated with an altered immune response. Additionally, PAD2 can translocate into nuclei and citrullinate histones, regulating gene transcription (10, 26).

Citrullination can change the net charge and increase the hydrophobicity of proteins, which subsequently alters the structures and functions of the proteins (35–39). The effects of citrullination are variable and debated. Hojo-Nakashima et al. revealed that PAD2 is beneficial as it catalyzes vimentin citrullination in THP-1 cells (a human monocytic cell line) to promote the differentiation and maturation of macrophages (40). By contrast, vimentin citrullinated by PAD2 is identified as an autoantigen in rheumatoid arthritis (RA), exhibiting the potentially detrimental role of PAD2 (32). Apart from vimentin, a large number of proteins are found to trigger autoimmune responses following PAD2-mediated citrullination (32). Interestingly, dysregulation of PAD2 activity has been implicated in many diseases such as RA (41), multiple sclerosis (MS) (42), and neurodegenerative disorders (43). Moreover, previous studies revealed that PAD2-catalyzed citrullination is an essential process during various modes of immune cell death, such as ETosis (13, 14) and pyroptosis (15). These modalities of immune cell death may play a major role in the pathogenesis of sepsis and other inflammatory diseases (44, 45). Consequently, it is critical to understand the role of PAD2 in host immunity and related diseases. In the following sections, the mechanisms *via* which PAD2 mediate cellular processes, regulate immune response, and cause diseases will be reviewed and discussed. Understanding the mechanisms of host immunity regulated by PAD2 may ultimately allow for design of novel therapeutic strategies for a multitude of immune disorders.

## PAD2 Expression in Immune Cells

### PAD2 in Macrophages

Macrophages are immune cells which exhibit relatively rich PAD2 expression (9). Macrophages play an important role both in innate and adaptive immunity. Phagocytosis and pyroptosis are two major pathways involved in the pathogen clearance by innate immunity (46–50). Macrophages contribute to adaptive immunity by presenting the antigens of pathogens to T cells (51–53). PAD2 can affect these immune actions through regulating the differentiation of macrophages (9, 40). Macrophages are derived from monocytes in circulation. Interestingly, although PAD2 mRNA can be detected in monocytes, it is not translated into PAD2 proteins until the initiation of differentiation (9). Moreover, a previous study revealed that the levels of PAD2 mRNA and proteins exhibit concomitant increases in THP-1 cells during the differentiation into macrophages (40). Nonetheless, the underlying mechanisms through which PAD2 mediate monocyte differentiation remain elusive.

**Figure 2:**
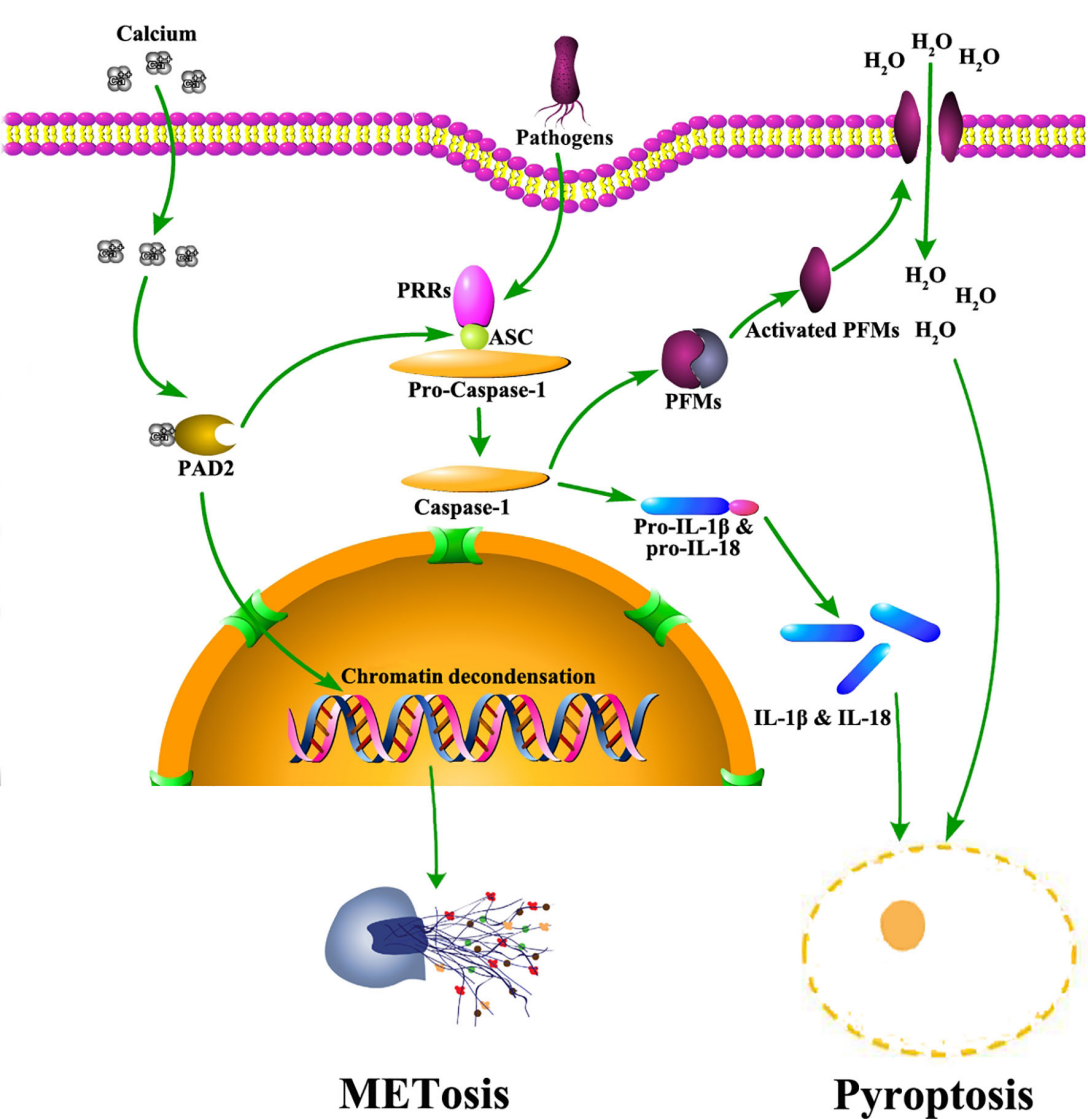
The role of PAD2 in METosis and pyroptosis in macrophages. Pathogens trigger calcium influx into cytoplasm of macrophages. Subsequently, PAD2 is activated due to elevated levels of calcium. Activated PAD2 translocates into the nucleus to induce histone citrullination and chromatin decondensation, leading to METosis. Also, PAD2 mediates pyroptosis *via* citrullinating ASC. Citrullinated ASC participates in the assembly of inflammasomes which activate Caspase-1. Caspase-1 facilitates the maturation of IL-1β and IL-18 *via* cleaving their precursors. Meanwhile, Caspase-1 cleaves and activates PFMs which insert into plasma membrane to create pores allowing massive water to flux in. As a result, macrophages swell and rupture to accomplish pyroptosis, releasing mature IL-1β and IL-18. ASC: apoptosis-associated speck-like protein containing a CARD domain; IL, interleukin; METosis, macrophage death with release of macrophage extracellular traps; PAD2, type 2 peptidylarginine deiminase; PFMs, pore forming molecules; PRR, pattern recognition receptor.

PAD2 also mediates the activation of pyroptosis (**Figure 2**), another important signaling pathway associated with anti-pathogen activities in macrophages (15). Pyroptosis is an inflammatory form of macrophage death induced by infection or chemical stimulation and mediated by Caspase-1 and/or Caspase-11 (54). Prior to the activation of Caspase-1, the stimulating signals are sensed by pattern recognition receptors, including NOD-like receptors and AIM2-like receptors, and initiate the assembly of inflammasomes (55–57). During the formation of inflammasomes, a quick increase of protein citrullination can be observed in macrophages (15). Specifically, ASC (apoptosis-associated speck-like protein containing a CARD), a critical component of inflammasomes, is also citrullinated. After PAD2 and PAD4 are dually suppressed by Cl-amidine, a pan-PAD inhibitor (58), the citrullination of ASC is reduced (15). Additionally, the activation of NLRP3 inflammasomes is also dampened, which subsequently diminishes macrophage pyroptosis. In agreement with these findings, our most recent experiments revealed that the knockout of *Pad2* in macrophages can decrease Caspase-1 mediated pyroptosis induced by *Pseudomonas aeruginosa* sepsis (PA-sepsis) (59). In contrast, *Pad4* depletion in the macrophages can increase Caspase-1 mediated pyroptosis in the mouse model of PA-sepsis (59). Therefore, PAD2-mediated ASC citrullination is probably a significant step during inflammasome assembly, which then regulates the activation of Caspase-1 and pyroptosis. Nonetheless, since little effort has been taken to explore the association between PAD2 and pyroptosis, the underlying mechanisms *via* which PAD2 affects Caspase-1 activation remain to be elucidated.

Aside from pyroptosis, macrophages are also reported to undergo another form of cell death termed METosis (**Figure 2**) (13), which describes the release of extracellular trap-like structures from macrophages (20, 60). Similar to NETs, Macrophage ETs (METs) are found in response to various microorganisms (61). METs are capable of trapping and immobilizing microbes to assist in microbial clearance (20). Several studies demonstrated that histone hypercitrullination catalyzed by PADs is an essential step during METosis (13, 62). Due to the alterations in net charges and structures, hypercitrullinated histones render chromatins more susceptible to decondensation (63). Most prior studies conclude that the process of citrullination is driven by PAD4, but a study by Mohanan et al. identified PAD2 as a major mediator in tumor necrosis factor (TNF)-α induced MET release from Raw264.7 macrophages (13). Therefore, further work is needed to clarify the association between PAD2 and METosis.

### PAD2 in Neutrophils

Overall, PAD2 seems to have minimal effects on neutrophils due to low expression. The distribution of PAD2 and PAD4 are different in neutrophils. Unlike in macrophages, the PAD that is predominantly expressed in neutrophils is PAD4 (64–66). PAD4 exists in granules, plasma membrane, and nucleus, while PAD2 is mainly detected in granules (64). Like macrophages, neutrophils can form NETs to defend against microbial infection (65, 67–69). NETosis also requires PAD-catalyzed histone hypercitrullination, which induces chromatin decondensation (63). In contrast to METosis, the citrullinating process in neutrophils is believed to be entirely mediated by PAD4 (65). However, our recent study found that selective inhibition of PAD2 can significantly decrease the generation of Citrullinated histone H3 (CitH3) in lipopolysaccharide (LPS)-stimulated neutrophils (70). The result suggests that PAD2 may also play a role in citrullinating actions within neutrophils. Furthermore, the extracellular release of PAD2 from neutrophils may still be able to citrullinate histone H3 and fibrinogen (64).

### PAD2 in T Cells

There are two major subtypes of T cells which are CD4+ T cells and CD8+ T cells (71). CD8+ T cells directly kill microbe-infected cells or tumor cells (72), while CD4+ T cells usually act indirectly to regulate immune response, thus coined “helper T cells” (Th) (73). The relationship between CD8+ T cells and PAD2 is not well studied, while several studies revealed that PAD2 can modulate the polarization and functions of CD4+ T cells (11, 74, 75).

The expression of PAD2 in naïve CD4+ T cells is much lower than that in memory CD4+ T cells, indicating that PAD2 may have effects on the differentiation of CD4+ T cells (74). Actually, the fate of differentiating CD4+ T cells is decided by two key transcription factors, GATA3 and RORγT (76). PAD2 can directly citrullinate these two transcription factors, which changes their DNA binding ability to modulate gene expression (11). PAD2 inhibition decreases the differentiation of Th17 cells but promotes the differentiation of Th2 cells from naïve CD4+ T cells (11). Reversely, PAD2 overexpression in human peripheral blood mononuclear cells reduces Th2 cell polarization and increases Th17 cell polarization (77). Meanwhile, PAD2 regulates the functions of CD4+ T cells (11). PAD2 deficiency enhances cytokine production in Th2 cells but suppress cytokine generation in Th17 cells. Interestingly, although PAD2 is not associated with Th1 polarization, PAD2 inhibition can impair interferon-γ production in Th1 cells (11).

In addition to directly altering the functions and polarization of CD4+ T cells, PAD2 can affect T cell activities by citrullinating certain chemotaxins (i.e., CXCL10 and CXCL11) that mediate the chemotaxis of T cells (34). T cells exhibit lower sensitivity to citrullinated CXCL10 and CXCL11. Therefore, fewer T cells will be attracted to inflammation sites, resulting in attenuated inflammatory response.

### PAD2 in B Cells

B cells are a subset of immune cells, which are responsible for antibody production and antigen presentation (78). The expression of PAD2 is low in B cells (79). Nonetheless, PAD2 is probably required for the transition of B cells to plasma cells, as the knockout of *Pad2* can cause a significant reduction in bone marrow plasma cells in a mouse model of TNF- α induced arthritis (80). Consequently, IgG produced by plasma cells is also decreased in *Pad2*^-/-^ mice, which is associated with alleviated severity of TNF-α induced arthritis (80). This may indicate that PAD2 is required for the development of plasma cells. However, given that PAD2-citrullinated proteins are antigens for B cells, another explanation may also be established: *Pad2* knockout reduces the generation of citrullinated proteins, thus resulting in decreased activation of B cells. Hence, further work is needed to clarify the role of PAD2 in B cells.

### PAD2 in Other Immune Cells

PAD2 can also interact with other cells to modulate immune response. For example, ATP upregulates the expression of Adamts-9, Rab6b, and TNFRII through activation of PAD2 in mast cells, contributing to the pathogenesis of RA (25). PAD2 and PAD4 inhibition by Cl-amidine also hampers functional maturation of dendritic cells induced by toll-like receptor agonists (81). As evidenced, there remains a paucity of studies exploring the interplay between PAD2 and immune cells. Further clarifying the mechanisms by which PAD2-mediated citrullination participates in immune activities can continue to advance the field in the future clinical applications of PAD2 guided therapies.

## PAD2 in Host Immunity

The immunomodulatory effects of PAD2 are mostly exerted by citrullinating key proteins involved in the cell signaling pathways. Thus, PAD2 may display different impacts on host immunity under different circumstances, which is determined by the roles of the citrullinated proteins in these pathways. The involvement of PAD2 in autoimmune diseases reflects its pro-inflammatory activity. The pathogenesis of RA is associated with elevated levels of PAD2-citrullinated proteins in synovial fluid (82). B cells can recognize the citrullinated epitopes and generate autoantibodies against the citrullinated proteins (83–85). In 70% of patients with RA, elevated levels of anti-citrullinated protein antibodies (ACPA) can be detected (86). After treatment with antirheumatic drugs, ACPA levels in circulation are significantly reduced correlated with decreased severity of RA (87, 88). These results suggest that protein citrullination by PAD2 can trigger an intensified inflammatory response in RA patients. Of note, RA patients who develop antibodies against PAD2 tend to suffer from less severe damage in joints and other organs (89). However, PAD2 sometimes exhibits the ability to inhibit inflammatory response. For example, Loos et al. reported that PAD2-mediated citrullination of CXCL10 and CXCL11 can reduce their chemotactic ability and thus result in diminished accumulation of inflammatory cells (34). PAD2 can also citrullinate certain transcription factors to mediate the differentiation of immune cells. The knockout of *Pad2* gene in mice can cause a shift in maturation of Th cells, which increases the differentiation of Th2 cells but decreases the differentiation of Th17 cells, rendering the mice susceptible to allergic airway inflammation (11).

The close association between PAD2 and host immunity is partly due to the relatively abundant expression in immune cells (74, 79). PAD2 functions as an important factor not only in the differentiation of immune cells, but also in several cell death signaling pathways (13, 15, 90, 91). Although PAD4 is identified to be the key regulator in NETosis (63, 92, 93), PAD2 may also play a part in the process as NETosis can still occur in the absence of PAD4 (94). Another type of cell death, pyroptosis, which mostly takes place in macrophages, is found to be regulated by PAD2 and PAD4 (15). Additionally, overexpression of PAD2 in Jurkat cells, which are derived from human T lymphocyte cells, can trigger enhanced apoptosis (91). Collectively, these findings indicate that PAD2 has an intimate relationship with immune cells and host immunity.

### Infections

#### Sepsis

Sepsis is characterized by a dysregulated inflammatory response that may result in multi-organ failure (95). The role of PADs in sepsis has been identified in some previous studies (70, 96–98). However, most of them explored the association between PAD4 and sepsis. This was probably due to the critical effects of PAD4 on NETosis, which is believed to be an important signaling pathway involved in the pathogenesis of sepsis (99). The application of pan-PAD inhibitors, which inhibit the activity of both PAD2 and PAD4, can remarkably improve the survival in mouse models of LPS-induced endotoxemia and cecal ligation and puncture (CLP)-induced sepsis (100–102). Nonetheless, when *Pad4*^-/-^ mice were used to explore the individual effects of PAD4 on sepsis, researchers found that *Pad4*-deficiency did not improve survival nor ameliorate bacteremia (94, 98). Accordingly, we revealed that a selective PAD4 inhibitor does not affect survival in LPS-induced endotoxic shock (70). Therefore, we began to hypothesize that the protective effects were derived from PAD2 inhibition. As expected, the employment of a selective PAD2 inhibitor in the same model of LPS-induced endotoxic shock significantly increased survival (70). Thereafter, our studies further demonstrated that the knockout of *Pad2* can improve survival in CLP-induced sepsis and PA-sepsis (59, 103). Therefore, it can be inferred that PAD2 likely acts as a critical mediator in the pathogenesis of sepsis.

**Figure 3:**
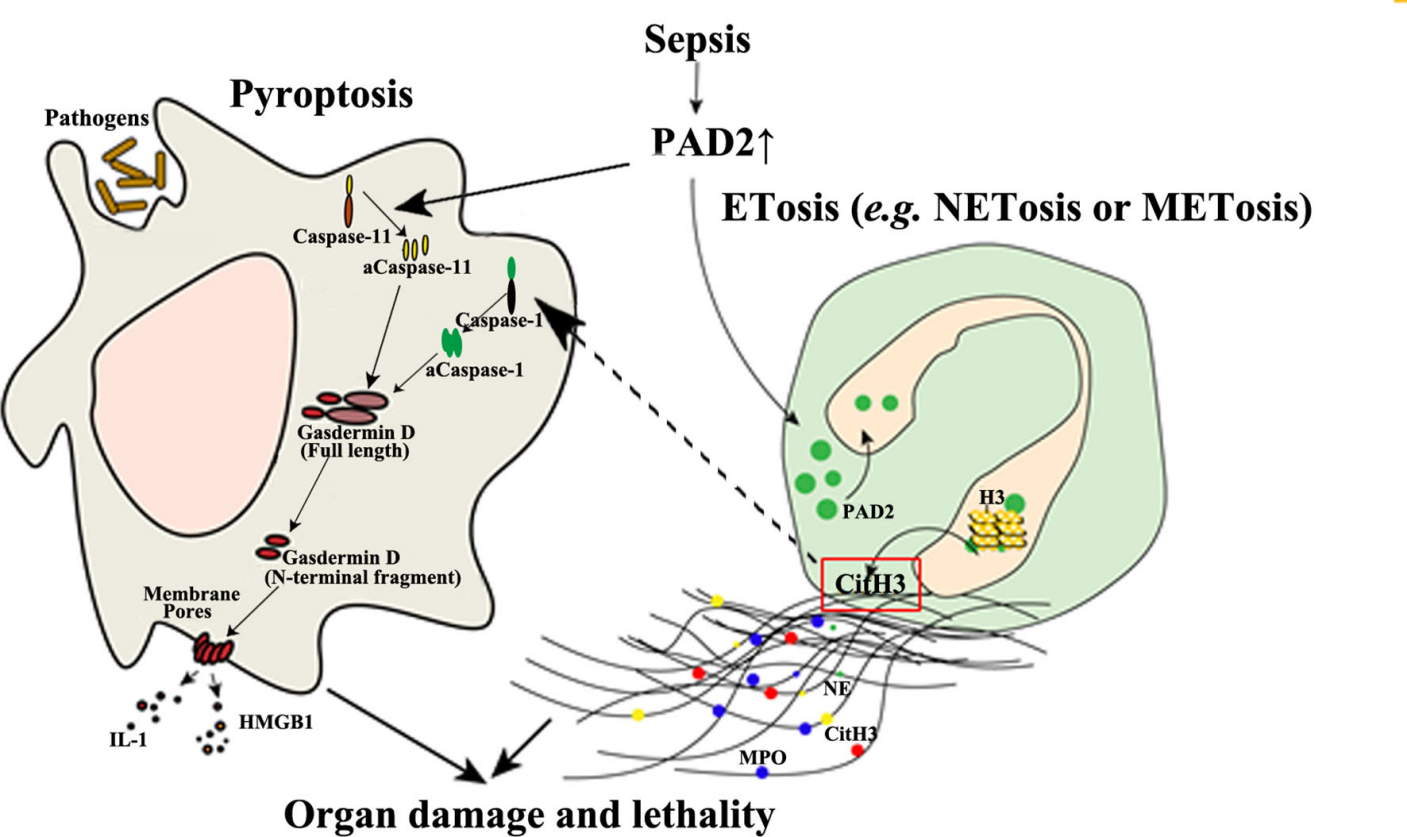
The detrimental effects of PAD2-mediated pyroptosis and ETosis during sepsis. PAD2 facilitates the activation of Caspase-11, a key regulator in non-canonical pyroptosis, and causes macrophage death. In addition, PAD2 can translocate into the nuclei of neutrophils or macrophages and citrullinate histone H3 to induce ETosis. CitH3 generated during this process may further activate the canonical pyroptotic pathway as a danger signal. aCaspase-1/11, activated Caspase-1/11; CitH3, Citrullinated histone H3; ETosis, cell death with release of extracellular traps (ETs); H3, Histone H3; HMGB1, high mobility group box 1; IL, interleukin; M/NETosis, neutrophil/macrophage death with release of extracellular traps; MPO, myeloperoxidase; NE, neutrophil elastase. Lines, pathways already known; Dotted lines, proposed hypothesis for the pathway to elucidated.

Given the minimal effect of PAD4 on sepsis, it raises questions as to why NETosis is closely related to sepsis and why septic animals can benefit from anti-NET therapies. The pathogenicity of NETs is derived from numerous components such as myeloperoxidase, DNA, and Citrullinated histone H3 (CitH3) (104). Such components are also found in extracellular traps released by other immune cells, such as METs, which are more likely to be mediated by PAD2, as PAD2 is more abundantly expressed in macrophages than PAD4 (61). “Anti-NET therapies” are referred to as the clearance of extracellular DNA or CitH3 (105–107), which also eliminate detrimental molecules from other sources including METs at the same time. In contrast, the knockout of PAD4 can only decrease the molecules coming from NETosis. This possibly explains why PAD4 inhibition is not protective during sepsis. In addition, we discovered that selective inhibition of PAD2 can decrease the release of CitH3 in neutrophils (**Figure 3**) (70). Furthermore, antibody neutralization of circulation CitH3, by a commercially available anti-CitH3 antibody, was not shown to attenuate endotoxemia (105). However, administration of the antibody recognizing CitH3 generated from both PAD2 and PAD4 significantly improved survival (105). These findings display the differing effects of PAD2 and PAD4 inhibition during sepsis.

In a mouse model of CLP-induced lethal sepsis, we have newly demonstrated that PAD2 protein is elevated in serum and lung tissue after CLP (103). In septic patients, serum concentrations of PAD2 are positively correlated to lactate (*r*=0.5, *p*=0.04) and procalcitonin (PCT) levels (*r*=0.67, *p*=0.003) (108). Since lactate and PCT are considered markers for the prognosis and the severity of sepsis (109, 110), elevated PAD2 levels in serum may also serve as a future clinical biomarker and predictor of outcomes. Circulating CitH3 was also found to be positively correlated with blood PAD2 (*r* values=0.0452, *p*<0.001) and PAD4 levels (*r* value=0.363, *p*<0.01), respectively (108). The levels of PAD2 in bronchoalveolar lavage fluid (BALF) from patients with sepsis and respiratory distress syndrome (ARDS) are also significantly increased compared with those in a healthy control group (108). Furthermore, the Pad2 gene was found to be over-expressed in cells of the BALF of patients with septic specific ARDS. The consistent findings support the possible usage of PAD2 as a biomarker for sepsis specific ARDS and may serve as a distinguishing factor between sepsis specific ARDS and other non-infectious causes of ARDS. PAD2 can mediate the onset of sepsis by directly regulating pyroptosis. We recently found that PA-sepsis induced pyroptosis in macrophages is dramatically decreased in the absence of PAD2, thereby attenuating acute lung injury and improving survival (59). In the murine CLP-sepsis model, *Pad2* depletion enhances bacterial clearance, attenuates sepsis-induced vascular permeability of lung and kidney, and improves survival (103). Moreover, we found that macrophages stimulated by LPS undergo diminished Caspase-11-dependent pyroptosis in the absence of PAD2, which can explain how *Pad2* knockout improves the outcomes of septic mice (**Figure 3**) (103). These findings have highlighted the detrimental role of PAD2-mediated pyroptosis in the pathogenesis of sepsis (**Figure 3**). PAD2 also catalyzes the generation of CitH3 which is recognized as a “danger” signaling molecule (70, 111, 112). Furthermore, it has been reported that “danger” signaling molecules (i.e., ATP and double strand DNA) can elicit the activation of pyroptosis *via* the Caspase-1 dependent pathway (113, 114). Based on this data, we hypothesize that CitH3 may play a role in activating the pyroptotic pathway and that PAD2 can also modulate pyroptosis in an indirect way. Altogether, PAD2 has the potential as both a biomarker and therapeutic target of sepsis.

Although we have demonstrated the effects of PAD2 activation on sepsis, the mechanisms by which PAD2 activation leads to these downstream effects in sepsis remain poorly understood. A previous study demonstrated that ATP induces PAD2 activity *via* P2X7 receptors (25). While ATP is required for almost all biological reactions as the universal energy source (115), once host cells are damaged, stressed, or infected by pathogens, intracellular ATP can be released to become extracellular ATP which serves as a key “danger” signaling molecule (116–118). Additionally, certain pathogens can also produce and secrete extracellular ATP (119, 120). The extracellular ATP may then bind to P2X7 receptors to induce calcium influx, leading to subsequent PAD2 activation (25). Nonetheless, there is limited evidence supporting that ATP release is responsible for the activation of PAD2 during infections. Thus, further work is required to elucidate the association between infection and PAD2 activity.

### Immune Disorders

#### Rheumatoid Arthritis

The manifestations of RA are characterized by chronic synovitis, systemic inflammation, and the generation of ACPA and rheumatoid factors (121). ACPA recognizes and binds to PAD2/4-citrullinated proteins, including vimentin, keratin, enolase, fibrinogen, and filaggrin (32, 122). ACPA can serve as a useful biomarker with high sensitivity and specificity, and is often a predictor of poor prognosis (123–126).

Among all the citrullinated proteins associated with RA, vimentin is the most frequently studied. Vimentin is an intermediate filament protein that plays a significant role in fixing the position of cytosolic organelles (127). Macrophages, which also express vimentin, are found in high levels in synovial fluid aspirates of RA joints (128). During calcium ionophore-induced macrophage apoptosis, vimentin is found to be citrullinated by PADs (90). Given the low expression of PAD4 in macrophages, PAD2 is likely the predominant PAD in the citrullination of vimentin. The cleavage of vimentin also occurs in the presence of calcium during macrophage pyroptosis (129). Since PAD2 is a calcium-dependent enzyme, it can be inferred that vimentin possibly undergoes citrullination prior to macrophage pyroptotic death. However, the mechanisms which citrullinated vimentin is associated with the pathogenesis of RA are not clear. One explanation is that the host loses its self-tolerance to citrullinated vimentin due to hereditary factors, which leads to production of ACPA (122, 130). As a result, massive ACPA-citrullinated vimentin complexes deposit in the joints, causing activation of complement systems leading to prolonged inflammation (131). Although genetic factors are closely related to the incidence of RA, the effects of environmental factors cannot be neglected (132). For example, a number of RA cases were found to be linked with infection (133). Thus, it is possible that infectious agent-induced macrophage death may be the initial step of RA onset. During the death processes of macrophages, vimentin is citrullinated by PAD2 and released. Meanwhile, more macrophages and other immune cells are attracted to the infected sites due to chemotaxis. Thereafter, citrullinated vimentin is recognized as an autoantigen which triggers the generation of ACPA. However, this hypothesis cannot explain the pathogenesis of ACPA-negative RA. Therefore, further work is required to understand the complexity of RA.

PAD2 can be detected in synovial fluid from RA patients (134). It was demonstrated that the major sources of PAD2 are inflammatory cells (8). RA patients with higher PAD2 levels in synovial fluid tend to have enhanced disease activity, suggesting that the level of PAD2 in synovial fluid is a potential prognostic indicator (135). Additionally, PAD2 can also be taken as an autoantigen by the host. RA patients who developed autoantibodies against PAD2 are likely to display attenuated joint inflammation and RA-related lung disease (89).

M1 macrophages, which are activated by the classical pathway, can secrete proinflammatory cytokines such as TNF-α and IL-1 and cause joint erosion. While M2 macrophages, which are activated by the alternative pathway, can produce anti-inflammatory cytokines (mainly IL-10 and TGF-β), contributing to vasculogenesis and tissue remodeling and repair, as recently observed in systemic sclerosis. Markers for both macrophage phenotypes may coexist on the same cell (136, 137). Recent studies have revealed that M1/M2 macrophage imbalance strongly contributes to osteoclastogenesis of RA (138). Eghbalzadeh et al. reported that NETs support macrophage polarization toward an M2 phenotype, displaying anti-inflammatory properties. PAD4 deficiency aggravates acute inflammation and increases tissue damage post- acute myocardial infarction, partially due to the lack of NETs (139). It remains largely unknown whether PAD2 affects macrophage polarization.

#### Multiple Sclerosis

MS is an autoimmune disorder in central nervous system characterized by chronic demyelination of nerve cells (140). Patients with MS usually suffer from loss of sensitivity, changes in sensation, difficulties in coordination or problems with vision (141). The effects of PAD2 on the pathogenesis of MS remain in debate. Researchers revealed that citrullination of myelin basic proteins (MBP) is increased in MS patients (42, 142, 143). Overexpression of PAD2 in mice leads to MBP hypercitrullination and myelin loss in central nervous system (144). Hypercitrullination will not only decrease the stability of MBP, but also put MBP at higher risks of being attacked by T cells (28, 75). Th17 cells, a subtype of T cell, shows enhanced reactivity to citrullinated MBP (75). As mentioned above, PAD2 can facilitate the polarization of CD4+ T cells into Th17 cells (11). Thus, PAD2 plays a critical role in MS pathogenesis. In line with these findings, a study demonstrated that PAD2 inhibition can attenuate disease severity in animal models mimicking MS (145). On the contrary, a study reported that deletion of *Pad2* gene in mice decreased levels of citrullinated MBP but did not reduce the incidence rate of experimental autoimmune encephalomyelitis (146). A recent study discovered that PAD2-mediated citrullination is indispensable during the differentiation and myelination of oligodendrocytes (147). Knockout of *Pad2* in mice will result in motor dysfunction and even decreased myelination in axons (147). Therefore, it is critical to keep a balanced PAD2 level in central nervous system as it maintains the normal structure and functions of nerve cells and further studies should continue to elucidate the role of PAD2 on MS.

### Cancers

Currently, PAD2 is implicated in skin tumors (148), breast cancer (10), colorectal cancer (149), and glioblastoma multiforme (150, 151). The intimate relationship between PAD2 and tumors is likely due to the role of PAD2 in modulating gene transcription. PAD2 is the only PAD that citrullinates arginine1810 (Cit1810) in repeat 31 of the carboxyl-terminal domain (CTD) of the largest subunit of RNA polymerase II (RNAP2) (152). Cit1810 is crucial for RNAP2 to overcome the pausing barrier close to the transcription start site, which enables the efficient transcription of highly expressed genes needed for cell cycle progression, metabolism, and cell proliferation (152).

The effects of PAD2 on the development of different tumors are not the same. For example, overexpression of PAD2 has been shown to augment the malignancy of skin tumors (153), while increased PAD2 expression has been linked to improved survival in patients with estrogen receptor (ER)-positive breast cancer (112). However, upregulated PAD2 expression in breast cancer is associated with resistance to tamoxifen treatment (154). These findings make PAD2 a mysterious modulator in tumorigenesis. In the pathogenesis of breast cancer and glioblastoma multiforme, PAD2 modulates gene transcription *via* citrullinating histones (112, 151). In colorectal cancer, however, PAD2 prevents tumor progression by citrullinating β-catenin thus inhibiting the Wnt signaling pathway. PAD2 inhibition will increase the sensitivity of breast cancer cells to tamoxifen (154), while the knockout of *Pad2* will induce great resistance to nitazoxanide in colorectal cancer cells (149). More work is needed to investigate the involvement of PAD2 in other tumors (149).

The therapeutic potential and applications of PAD2 in cancer remains to be further clarified. However, given the role of PAD2 in tumorigenesis and response to chemotherapy, PAD2 will continue to be a biomarker and target of continued interest in the era of personalized cancer care.

## Conclusions and Future Perspectives

**Figure 4:**
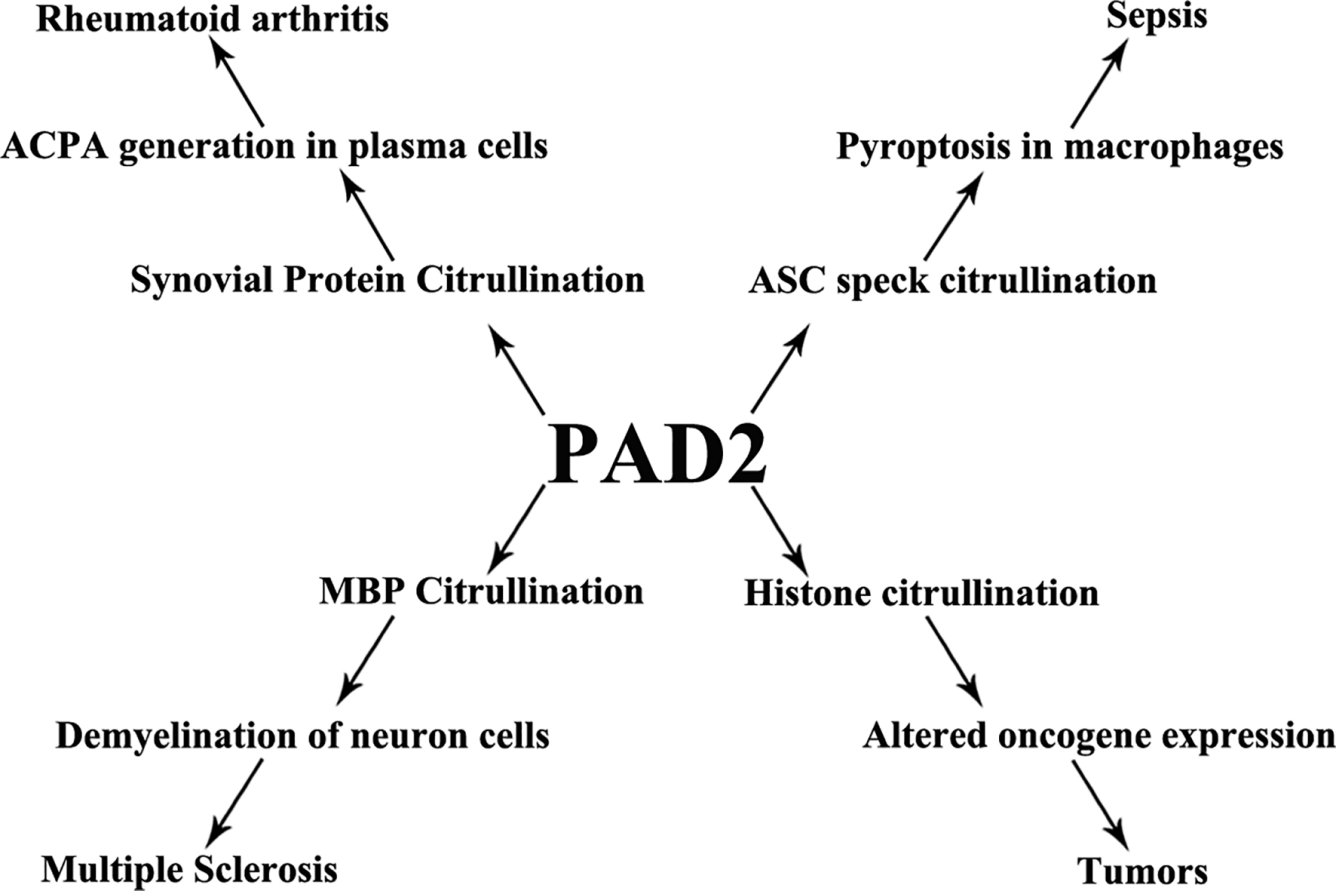
PAD2 and immune disorders. A schematic view showing that PAD2 is associated with a number of diseases and the possible mechanisms underlying pathogenesis. ACPA, anti-citrullinated protein antibodies; ASC, apoptosis-associated speck-like protein containing a CARD domain; MBP, myelin basic protein.

Citrullination is a posttranslational protein modification catalyzed by PADs and is involved in host immunity. PAD2 has wide-reaching roles through its citrullination of a variety of target proteins. Dysregulated activity of PAD2 is associated with a series of immune disorders including sepsis, RA, MS, and tumor formation (**Figure 4**). In this review, we have summarized PAD2 specific functions on cell death control, transcription regulation by citrullination of arginine 26 on histone H3 (*e.g.*, sepsis, tumor), and citrullination of vimentin (*e.g.*, RA). We highlight several citrullinated proteins to demonstrate the contributions of PAD2-mediated protein citrullination to RA, sepsis, and cancer within each specific environment. Given that CitH3 is also found to be a biomarker in patients with cancers (155, 156), more epigenetic studies are needed to explore if and how citrullination of histone H3 interferes with transcription factors to regulate RA, sepsis, and cancers. We propose that PAD2 is a promising novel biomarker and therapeutic target for a broad spectrum of diseases including autoimmune and inflammatory diseases, sepsis, MS, and several types of cancer.

## Author Contributions

ZW and YL drafted the manuscript. PL, YT, WO, JH, HA, and YL made significant revisions to the manuscript. All authors contributed to the article and approved the submitted version.

## Funding

This work was funded by grants from the National Institute of Health R01 (Grant# RHL155116A) to YL and HA, and the Joint-of-Institute (Grant# U068874) to YL.

## Conflict of Interest

The authors declare that the research was conducted in the absence of any commercial or financial relationships that could be construed as a potential conflict of interest.

## Publisher’s Note

All claims expressed in this article are solely those of the authors and do not necessarily represent those of their affiliated organizations, or those of the publisher, the editors and the reviewers. Any product that may be evaluated in this article, or claim that may be made by its manufacturer, is not guaranteed or endorsed by the publisher.
